# Novel Engineered Materials: Epoxy Resin Nanocomposite Reinforced with Modified Epoxidized Natural Rubber and Fibers for Low Speed Wind Turbine Blades

**DOI:** 10.3390/polym13162761

**Published:** 2021-08-17

**Authors:** Chainuson Kasagepongsan, Sunisa Suchat

**Affiliations:** 1 Renewable Energy and Environmental Research for Local Community Unit (REERCU), Faculty of Science and Technology, Suratthani Rajabhat University, Surat Thani 84100, Thailand; chainuson.kas@sru.ac.th; 2 Faculty of Science and Industrial Technology, Prince of Songkla University, Surat Thani Campus, Mueang, Surat Thani 84100, Thailand

**Keywords:** nanocomposites, accelerated weathering, vertical axis wind turbine blade, low wind speeds, Savonius, Darrieus

## Abstract

The objective of this study was to investigate nanocomposite materials with good outdoor resistance for wind turbine blade application. The nanocomposites based on epoxy resin with 5% of epoxidized natural rubber (ENR 50), 3% of nanofiller, and glass fibers, were subjected to experiments. The weathering resistance of nanocomposites was evaluated from the change in mechanical properties caused by accelerated aging, induced by UVB radiation in a weathering chamber. The accelerated aging improved tensile strength by about 35% at 168 h of exposure to UVB, via a curing effect. The nanocomposites were optimized for all the parts of wind turbine blades (Savonius and Darrieus types) that are generally designed for high strength, low weight, weathering resistance, and low rotational speed (2 m/s). A tree wind turbine with nanocomposite blades produced 5 kW power output when tested. Based on the findings in this work, the innovative nanocomposites have potential in manufacturing wind turbines to generate electricity.

## 1. Introduction

Currently, the use of fossil fuels is associated with pollution and resource depletion that present important challenges, which demand reduction of emissions and use of renewable energy sources [1]. The total amount of wind energy on the planet is vast and, during the last several years, commercial turbines have been installed all over the world. Wind energy has attracted significant attention in the developed countries in Europe, more so than other renewable forms of energy [2,3]. The advantages of wind energy are cleanliness and negligible safety issues, and this is one of the fastest-growing energy resources in the world [4], ensuring a ready supply of wind turbine equipment [5]. Wind turbines are primarily used to capture wind energy for the purpose of generating power [6,7]. Generally, the wind turbines fall into two categories: horizontal and vertical axis turbines. There are two types of vertical axis wind turbines, namely with Savonius or Darrieus rotors, which offer the advantage that the wind turbine depends only on the drag force. A vertical-axis wind turbine has the main rotor shaft transverse to the wind direction, while the main components are located at the base of the turbine [8,9]. The vertical axis wind turbines with Savonius and Darrieus rotors operate at low wind speeds. Achieving the goals in green energy adoption will require continuous advances in small wind turbine technology, progressive improvements in small turbine manufacturing, efficient nanomaterials for blades, and techniques suited for small wind turbine technology opportunities in Thailand. This work seeks to generate energy from renewable resources, with the value of wind power in being a low-cost, clean source. There are significant new business opportunities for manufacturing and material innovation. Savonius and Darrieus (S-D) is a type of vertical-axis wind turbine [10] mixing the two common designs, including a turbine mounted to the rotating element (Savonius rotor model) [11] and a smaller model that looks somewhat like a beater (Darrieus rotor model) [12]. Savonius models are more common and let air in through a hub to turn a generator; the turbine is driven by rotational momentum of air [13,14]. Attention has been directed to the Savonius and Darrieus wind turbines that offer simple design, self-starting capability, compact size, and easy installation and maintenance [15]. Furthermore, they operate independently of wind direction. Because low wind speeds are common in some countries where these wind turbines are available, it is necessary to design the turbines to be effective at low wind speeds in generating electrical power [16,17]. However, the critical element is the blade that converts the wind to mechanical power. A combined Savonius wind turbine has been reported to have a maximum power coefficient of 0.4 with a required start-up torque rated at more than 0.1 Nm at low-speed winds of 2 m/s [14,15]. The Darrieus-Savonius blade performance is affected by configurational conditions, geometry, and air flow parameters, and the number of blades also affects the power efficiency [7,10]. The wind turbine blade is a critical component made of polymeric composites. These composites can include carbon fiber, glass fiber, nylon, and stainless titanium, because these are strong but light-weight [18,19]. Up to 50% of European wind blade manufacturers nowadays use epoxy resins due to their excellent properties, such as light weight, good adhesion, high modulus, resistance to fatigue, low creep, reasonable elevated temperature performance, and lack of shrinkage after cooling [20,21,22,23,24]. However, they are brittle and easily fail under impact. [25,26]. Other materials have been added to the epoxy resin to improve toughness, such as inorganic glass particles [27,28]. Addition of elastomers and thermoplastics has also been studied, and the mechanical properties depend on the rubber portion in the mixture [18,20]. It was suggested that the impact strength of epoxy resin can be improved by blending in epoxidized natural rubber, ENR. A prior study found that ENR 50 (with 50 mol% substitution of epoxide groups) offered better impact strength than ENR 25 [29]. Therefore ENR 50 could be considered for improving the impact strength of epoxy composite [18,30] for application in wind turbines, specifically in the blades. Environmental aging is caused by various agents, such as humidity, wind, and ultraviolet radiation (UV), and consists of irreversible changes in the molecular structure that affect durability. With the ever-increasing use of these materials, aging has become a concern to the aeronautic industry and manufacturers [31,32]. In a previous study, improvements in the mechanical properties were attributed to post-curing reactions [33,34]. However, the post-curing reactions have significant effects on the composite properties, potentially including weakening of the fiber-matrix interfaces [34,35,36]. The design requirements on material properties namely high strength and low density are needed to reduce gravity forces and to minimize the cost of power. The prior studies are limited in assessing the effects of aging or the extent of aging effects. An objective of this current study was to assess the weathering resistance of nanocomposites for wind turbine blade applications, when exposed to alternating cycles of accelerated weathering by exposure to UVB radiation and water condensation. The investigation was motivated by Savonius and Darrieus wind turbines operating at low wind speeds of 2 m/s.

## 2. Experimental

### 2.1. Materials and Methods

The renewable ENR 50 (epoxidized natural rubber latex) was synthesized by the same method as in a previous study [18]. The nanocomposite in liquid state has advantages in the wind turbine blade preparation. The liquid state namely enables easy mixing and good dispersion of ENR in nanocomposite and the epoxy also reduces number of processing steps. The epoxy resin (Epicholrohydrin–bisphenol A) was purchased from Aditya Birla Chemical Industry Co., Ltd., Bangkok, Thailand. The curing agent (polyamide resin) was supplied by Siam Chemical Industry Co., Ltd. Bangkok, Thailand. The 40% colloidal nanosilica in the form of white nanosilica powder, surface-modified with methyl triethoxy silane [20], was supplied by Boss Oftical Co., Ltd., Bangkok, Thailand. The most commonly used types of composite materials in the wind turbine are glass fiber reinforced. E-glass (EMC 100) chopped strand mat was purchased from Neotech Composite Co., Ltd., Bangkok, Thailand. Glass fibers are thin strands of silica-based glass. The stiffness of composite is determined by the stiffness of glass fibers and their volumetric content. Typically, the glass fiber and epoxy composites for wind blades have high strength with heat resistance and corrosion resistance.

### 2.2. Sample Preparation

The epoxy resin nanocomposites were loaded with various weight fractions (1–5 phr: parts per hundred of rubber, i.e., mass percentage relative to the rubber) of nanosilica filler, then mixed with a stirrer. In the first step, the mixer (5 phr ENR 50, nanosilica, and epoxy resin) was run at 50 rpm speed for 15 min at 30 °C (the ambient room temperature). Then, 35 phr of the polyamide resin was added and stirred for 5 min. To conclude, the nanocomposite samples were prepared by casting in molds. At least three replicates of each sample type were used in each test.

### 2.3. Characterization

FTIR was spectra of materials were recorded (Shimadzu, Japan) based on 32 scans over the range 400–4000 cm^−1^ in ATR mode.

### 2.4. The Mechanical Properties of the Nanocomposite and Wind Turbine Blade Samples

The Izod impact strength of cured nanocomposites specimens used notched samples measured according to ASTM D256. The specimen dimensions were 63.5 × 12.7 × 3.0 mm^3^ and the depth under the notch of the specimen was 10.2 mm. A universal testing machine (Lloyd LR 10 K, Lloyd Testing, West Sussex, United Kingdom) was used to measure the tensile properties of the nanocomposites according to ASTM D638, with Type I specimens of 50 × 3 mm^2^. The hardness (Shore D) was tested according to ASTM D2240. The nanocomposite specimens used were at least 6.0 mm thick. The hardness (Barcol) was tested according to ASTM D2538 for the fiber-nanocomposites. Flexural strength was determined according to ASTM D790 for all samples, with the aspect ratio 16:1. The specimen dimensions were 127 × 12.5 × 3 mm^3^ at 50 mm/min. The values reported are averages of five specimens.

### 2.5. Morphology

The SEM inspection of morphology was performed for fracture surfaces at the notch, in order to assess the uniform failure mode. The specimens were sputter coated with gold before imaging, and, also, an energy dispersive X-ray (EDX, SEM-EDX, CAE’s Austin trading operation, TX, USA) analyzer was used to examine the composition from EDX spectra.

### 2.6. Thermal Properties

Thermal properties were determined by thermogravimetric analysis (TGA, Eltra GmbH Inc., Haan, Germany) over the temperature range 30–900 °C under N_2_ and O_2_ flushing at separate stages with a heating rate of 10 °C min^−1^ and a gas flow rate of 100 mL/min. The accelerated aging tests followed ASTM G154 cycles I and II using a QUV test LU-0819, Q-panel lab products, MA, USA. The conditions were: UVB radiation at 340 nm and 1.55 W/m^2^ at 60 °C for 8 h, water spray on the specimen for 15 min, and water-condensation at 50 °C for 4 h. The total aging time tested was 168 h (i.e., one week).

### 2.7. Forming the Wind Turbine Blades

The application of nanocomposite to forming the vertical axis wind turbine blades was performed with the hand lay-up technique. Hand lay-up is the most common and least expensive open-molding method, because it requires the least amount of equipment. Reinforcements in the form of glass fibers are cut to fit the mold size and placed on surface of the mold. Then nanocomposite in liquid form is mixed thoroughly in suitable proportions with a curing agent (TH 7255 hardener) and poured onto the surface of fiber mat already placed in the mold. The nanocomposite is uniformly spread with a brush. A second fibrous mat layer is then placed on the nanocomposite surface and a roller is moved with a mild pressure on the mat to remove any air trapped under it, as well as to squeeze out any excess nanocomposite. The process is repeated for each fiber mat layer, until the required number of layers is stacked. A release wax gel is sprayed on the inner surface of the top mold plate, which is then placed on the stacked layers and pressure is applied. The mold is then cured for 24 h, at the ambient room temperature (30 °C), and the nanocomposite part is taken out and further processed.

**Figure 1:**
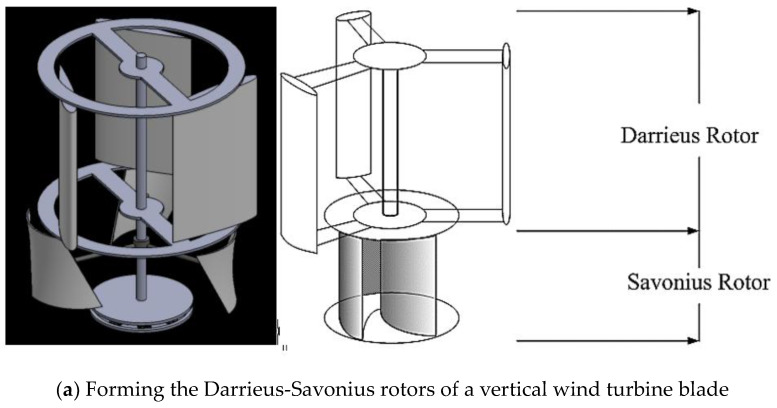

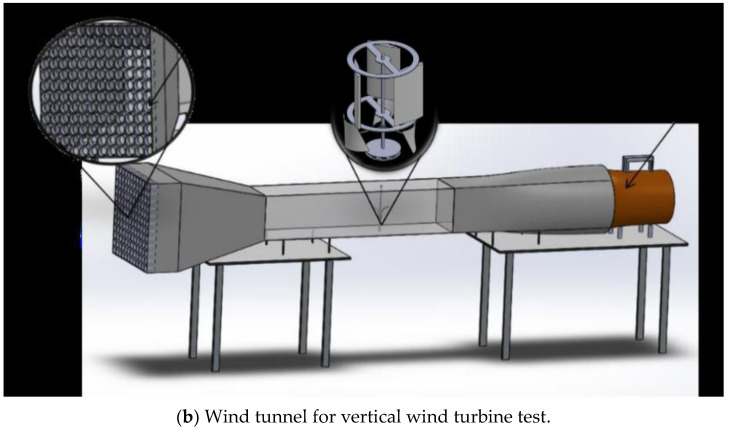
A vertical wind turbine blade section (**a**), and the wind tunnel test (**b**).

The design of a vertical wind turbine blade in a Savonius-Darrieus rotor used the nanocomposite (the modified epoxidized natural rubber and nanosilica combined with glass fiber and epoxy resin) with twisted buckets, while interference of shaft made use of stainless steel twisted buckets and interference of shaft types were used to study the properties of the blade in Figure 1a. Figure 1b shows the wind tunnel. Experiments were conducted at the facilities of the Prince of Songkla University in Thailand, where the wind tunnel was made of iron sheets. The wind tunnel was driven by an inverter-controlled AC. The test had a length of 2.4 m and offered a 1.2 m × 1.2 m cross-section. The maximum velocity generated reached only low speeds (4 m/s), but these experiments were carried out in the low range of wind speeds at 1.2 m/s. This study measured the flow field around the wind turbine with an anemometer, and a Digiton Photo Tachometer model DT-2234C (A Digiton Photo Tachometer, Finedayplus Co., Ltd, Nonthaburi, Thailand) with accuracy of 0.1 rpm was used to record the rotational speed.

## 3. Results and Discussion

### 3.1. Characteristics of the Samples

#### 3.1.1. Fourier Transform Infrared Spectroscopy (FTIR)

**Figure 2:**
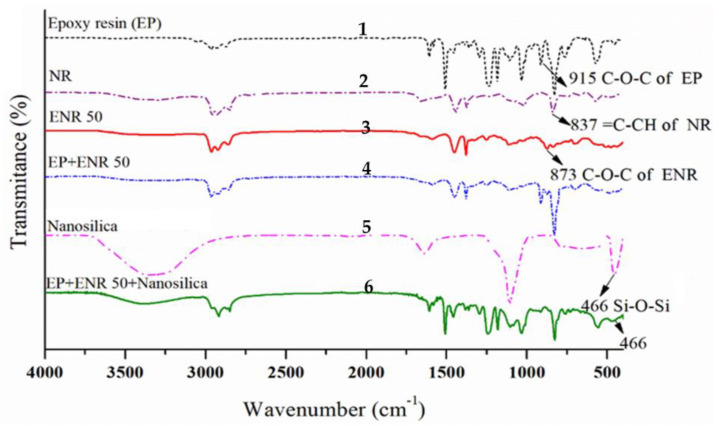
FTIR spectra of nanocomposite and its main raw materials: 1: epoxy resin (EP), 2: natural rubber (NR), 3: Epoxidized Natural Rubber (ENR 50), 4: EP + ENR 50, 5: nanosilica, and 6: EP + ENR 50 + nanosilica.

The chemical structure of nanocomposite from epoxy resin, ENR 50 and nanofiller was confirmed in Figure 2 and Table 1. In Figure 2, line 1 shows epoxy resin that gave characteristic band at 915 cm^−1^ for C–O deformation. The C=C bonds in NR were randomly converted into epoxide groups, so new characteristic peaks emerged at 1251 and 873 cm^−1^ (C-O-C, asymmetric stretching) in the spectrum of ENR 50 (lines 3 and 4) [37]. The nanosilica shown in lines 5 and 6 had three main characteristic peaks of silica in the range from 466 cm^−1^ to 1300 cm^−1^ [38].

#### 3.1.2. Particle Size of Nanofiller

**Figure 3:**
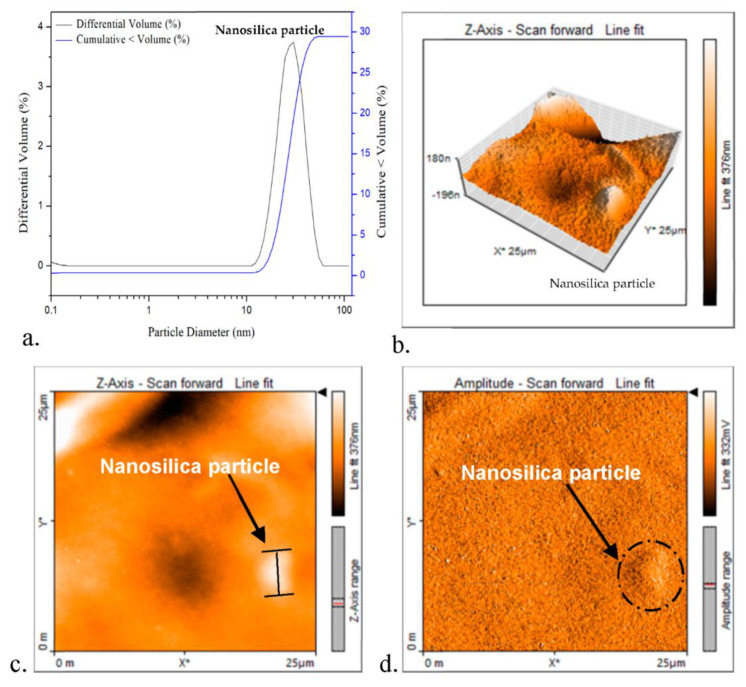
The particle size of nanosilica by DSL and AFM images: (**a**) particle size distributions by DSL, (**b**) 3-D topography images, (**c**) 2-D phase, and (**d**) nanoparticle phase separation by AFM technique, * in the figure as X axis scale or Y axis scale.

The particle size of nanosilica can also be confirmed by Laser diffraction and AFM techniques, shown in Figure 3. The particle size distributions for nanosilica are shown in Figure 3a. Dynamic light scattering (DLS) delivers rapid, precise, and repeatable nanoparticle size data and it is an essential tool for the nanoparticle technologist.

The z-average diameters obtained with the SZ-100 are listed. The size distribution of nanosilica was unimodal with one peak. In nanosilica particles, about 90% of volume was particles larger than 50 nm in diameter, and 10% were smaller than about 35 nm; the average diameter of particles (weighted by volume) was around 43 nm.

The particle size of nanofiller can be confirmed by AFM (atomic force microscopy) in Figure 3b–d that show topographies with nanofiller in 2-D and 3-D images, and these clearly allow distinguishing nanoparticles. The average nanosilica particle size was estimated as 43 nm, by image analysis. The nanosilica reinforcing filler size in the composite material is important.

#### 3.1.3. SEM/EDX

**Figure 4:**
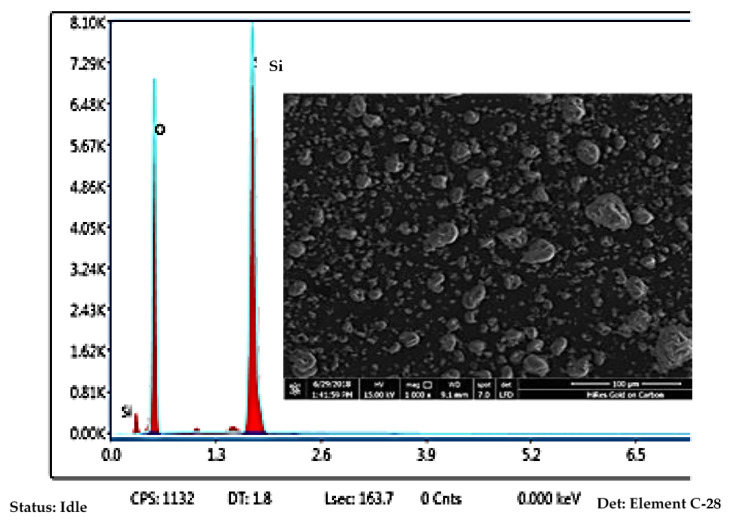
SEM-EDX analysis of the nanosilica.

SEM/EDX analysis also provides chemical composition of the nanosilica as illustrated in Figure 4.

Figure 4 shows SEM-EDX results for nanosilica. It can be seen that the chemical components in nanosilica are mainly silicone (Si) and oxygen (O), which matches the nanosilica structure with a high 58.77% weight percentage of Si and 41.23% of oxygen by weight. This type of nanosilica is very purified and has high contents of silicone and oxygen, and its purpose here is to reinforce with improved impact strength the rubber nanocomposite.

### 3.2. The Mechanical Properties of Nanocomposite Samples

Table 2 shows the mechanical properties of nanocomposites with several alternative loadings (0–5 phr) of 40% nanosilica and 5 phr of ENR 50 per 100 phr of epoxy resin. Generally, it is reasonable to perform this check with nanocomposites intended for applications where inertial or centrifugal forces can be important. The mechanical properties of nanocomposite samples, including impact strength, tensile strength, hardness, and density, were also tested.

**Figure 5:**
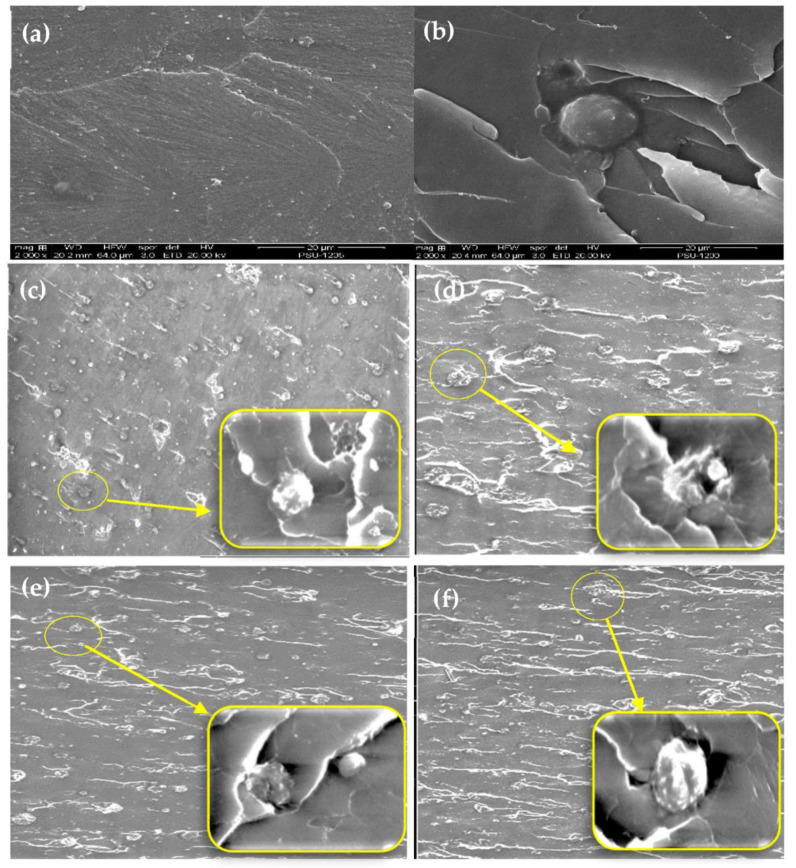
SEM micrographs of; (**a**) epoxy resin, (**b**) epoxy resin/5 phr ENR 50, and nanocomposite with nanosilica, amounts 1–4 phr (**c**–**f**).

For significantly improved mechanical properties, nanosilica loadings of 0-until 5 phr were tested to optimize the amount of nanosilica in the nanocomposite. The mechanical properties indicate partial compatibility, added rubber can improve the impact resistance by dissipating energy in the epoxy-rubber blend. In addition, the impact strength of nanocomposite, results from the notched Izod impact tests are displayed in Table 2. The amount of energy absorbed in the test is the energy required to cause fracture, and the impact resistance is displayed in Figure 5. As can be observed, the impact strength was improved by blending in nanosilica, and it was maximal at 3 phr nanosilica content. This improvement might be due to the dispersed rubber inclusions in epoxy absorbing energy. The rubber phase increased the viscosity of the epoxy mixture and reduced cross-link density and tensile strength. The high reinforcing efficiency of layered nanosilica in rubber composites, even at low nanosilica loadings, is largely due to good nanoscale dispersion (Figure 4 and Figure 5). Regarding, the nanosilica was contributes to hardness of the composite, progressively increased with nanosilica loading. This is due to nanosilica hindering the molecular mobility of epoxy resin, thereby increasing hardness, and effected to the loss of rubber elasticity reduced resistance to tearing consistently with nanosilica loading, while the reinforcing filler nanoparticles increased hardness. Whereas, the hardness of nanosilica loaded composites was 78 to 81 Shore D, not so much significantly different from the baseline case. The density of the nanocomposites is shown in Table 2. Density increased slightly with nanosilica loading but without significant difference, only marginally without affecting suitability for blade applications.

### 3.3. Morphology of Nanocomposites

SEM micrographs of notched Izod impact fracture surfaces, in Figure 5a for neat epoxy resin, and in Figure 5b for 5 phr ENR 50 blend with epoxy resin, while nanosilica is seen in Figure 5c–f. The micrograph of epoxy nanocomposite shows smooth and glossy exterior with numerous wavy cracks. Figure 5e show the blend of EP/ENR 50/3 phr nanosilica with superior interactions at phase boundaries, which gave the highest impact strength. It should be noted that adding more than the 3 phr nanosilica in nanocomposite can worsen the mechanical properties.

The mechanical properties improved with nanofiller loading. The dispersed nanosilica in epoxy composite improved the mechanical properties. Reinforcing filler in nanoscale, small particle sizes with high surface area have good adhesion potential, with the ability to adhere and disperse in good and can mix well. An experimental design is given, to develop composites, it is necessary to have some basic understanding of dispersion and toughening of these composites. Improved dispersion resulting in blending for good of the interface adhesion between silica particles and ENR rubber-liked materials reinforced epoxy. Lanna [20] reported the interface adhesion is high from the matrix structure of the composite will be similarly (some part), silica, rubber-liked materials reinforced epoxy resin. Moreover, epoxy resin and rubber are the coated materials as adhesives, can enhance the interfacial adhesion and demonstrates that can lead to a good interface layer, which is detailed of particle size, particle/matrix interface adhesion and particle loading on the strength and toughness of composites are reviewed.

Increasing the nanofiller loading level continuously improved strength and fracture toughness. The 3 phr nanosilica loading in the mixture was the best choice, with compatibility and high strength, and without significant negative effects.

### 3.4. Aging Effects on Thermal and Mechanical Properties of Nanocomposite Samples

#### 3.4.1. The Mechanical Properties of Nanocomposites

**Figure 6:**
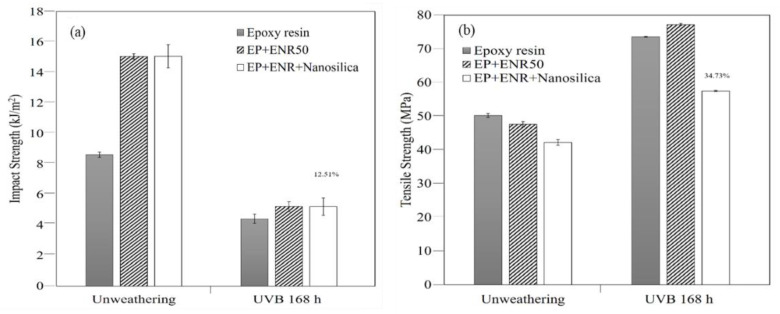
The mechanical properties of nanocomposites after UVB exposure for accelerated aging: (**a**) impact strength and (**b**) tensile strength.

**Figure 7:**
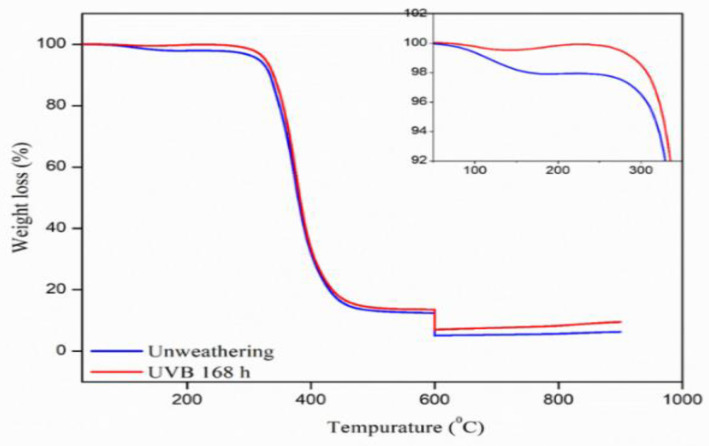
TGA thermograms of nanocomposite samples.

The aging effects on thermal and mechanical properties of nanocomposite samples were determined. The mechanical properties before and after accelerated aging of the optimal nanocomposites are shown in Figure 6 and Figure 7. The specimens were tested for weathering resistance by using the accelerator chamber for 168 h with UVB exposure. After accelerated aging, it was found that, the impact strength of nanocomposites had decreased, as can be seen in Figure 6a, according to the Izod impact test. The nanocomposite was post-cured by the UV increasing crosslinking, so that the overall hardness increased. The Izod pendulum impact test indicates absorbed energy at the breaking point of the specimen. The amount of energy it took to break composite specimens of specified size was measured based on ASTM D256. Figure 6b shows that accelerated aging improved tensile strength in agreement with a prior study [15]. Effects of accelerated aging with UVB treatment for 168 h were evaluated. The nanocomposites had good strength properties after accelerated aging.

This is due to the small particle size of nanofiller and of ENR 50, that both had good compatibility and dispersion in the nanocomposites. However, the disadvantage of the sample showed color change from polymer degradation.

Nanocomposite approach to engineered biobased ENR 50 with epoxy resins and nanofillers fits the search for nonpetroleum-based structural materials, with high performance and light weight.

#### 3.4.2. The Thermal Stability Investigation

Thermogravimetric analysis (TGA) can be used as an indicator of thermal properties. The thermal properties before and after the weathering by accelerated aging, are shown in Figure 7 (TGA thermograms of nanocomposites).

The initial decomposition at approximately 200 °C is thought to be the decomposition of natural rubber epoxide molecules, and this decay behavior was clearly observed in the nanocomposites. The decomposition temperature from the TGA curve and the maximum degradation temperature (Tm) was approximately 370 °C. The above decomposition was the decomposition of the epoxy resin, and the residual content was approximately 6–7% as shown in Figure 7, clearly showing that after weathering the nanocomposite showed better stability against heat than without weathering. As a result of increased crosslinking density, the nanocomposite had improved thermal stability. At 600 °C temperature, the nanosilica in the composites might be partly decomposed in the two steps and some of the residue would still remain, and its amount tended to increase with nanosilica loading. TGA was performed from 600–900 °C under oxygen atmosphere, while the initial part was run with nitrogen flushing. In the latter part there was an immediate decomposition step.

#### 3.4.3. Mechanical Properties of Wind Turbine Blades from Nanocomposite-Fiber Samples

− *Impact strengths of nanocomposite-fiber samples*

**Figure 8:**
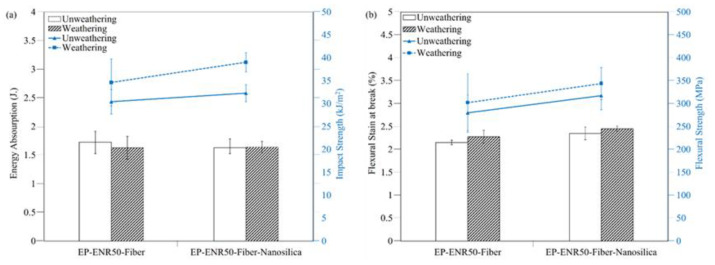
The mechanical properties of wind blade nanocomposites after accelerated aging: (**a**) impact strength and (**b**) flexural strength.

The nanocomposite contains 100 phr epoxy resin, 35 phr curing agent, 5 phr ENR 50, 3 phr nanosilica, and 3 layers’ glass fiber. Wind turbine blades were formed from the nanocomposite-fiber samples. Mechanical properties were determined after weathering in the accelerator chamber for 168 h using UVB exposure. Accelerated aging improved the impact strength of the nanocomposites markedly, see Figure 8a.

This is due to epoxy resin curing caused by UV radiation, which increased crosslinking and hardness. It was found that adding nanosilica gave higher energy absorption capacity to the epoxy resin. This is due to nanosilica crystallites causing holes or gaps that can absorb mechanical energy. The loss of energy during impact is the energy absorbed by the nanocomposite specimen during impact. The nanocomposites had higher impact strengths than neat epoxy. The wind blades from nanocomposite with glass fiber had increased hardness, but without significant difference. In rotor blade property requirements, high strength is needed to withstand fatigue and decrease degradation during use, high stiffness is needed to maintain aerodynamic shape of the blades, to prevent collisions, and low density is needed to reduce gravity forces and to minimize the cost of power.

− *Flexural strength of nanocomposite-fiber samples*

Figure 8b the flexural strengths of weathered and unweather of epoxy samples were 290 MPa, while the corresponding value was 345 MPa with nanofiller added. It appears that the addition of nanofiller into the specimens enhanced their overall flexural strength, which could be related to dispersion and compatibility of the nanofiller.

The interlamina fracture toughness is an important and to considered that a review of a previous study ([38,39], that using SEM, and tensile testing can be used instead of interlaminar fracture toughness of a glass fiber (GF)/composite were investigated. SEM observations, that the modified composites formed between ENR 50, GF, and epoxy matrix exhibited improved interfacial adhesion and improvements in tensile strengths. The interlaminar region of a laminate, GF fiber bridging at the crack path deviated from the GF/epoxy resin layers without undergoing any disbonding. The fracture toughness of epoxy resin composite was induced by the ENR 50 globular nodules attached to the epoxy matrix improving the impact strength. The rubber and epoxy resin interfacial interactions are improved. The potential improvements obtained by incorporating these toughening agents could increase the damage tolerance of GF/nanocomposites, which are extensively employed where corrosion resistance is critical such as in wind turbine blades.

### 3.5. Application to Blades of a Vertical Wind Turbine

The blades of a vertical wind turbine were for Savonius and Derrieus (S-D) rotor type designed to operate at low wind speeds of around 2 m/s. The epoxy resin was blended with ENR 50 and nanosilica for improved impact strength, required in wind blade applications. The low-speed wind turbine is very interesting for harvesting energy with a wide range of applications. S-D are vertical wind turbines that can be operated with low complications. Lighter materials used for producing the blades can reduce the inertial forces, leading to faster start-up of the wind turbine. Darrieus (H-type) was the upper unit while Savonius (Interference of shaft type) was the lower unit in a vertical turbine fabricated using the nanocomposite.

#### 3.5.1. Forming a Wind Blade with Hand Lay-Up Technique

*Wind Blade Structure Design*

**Figure 9:**
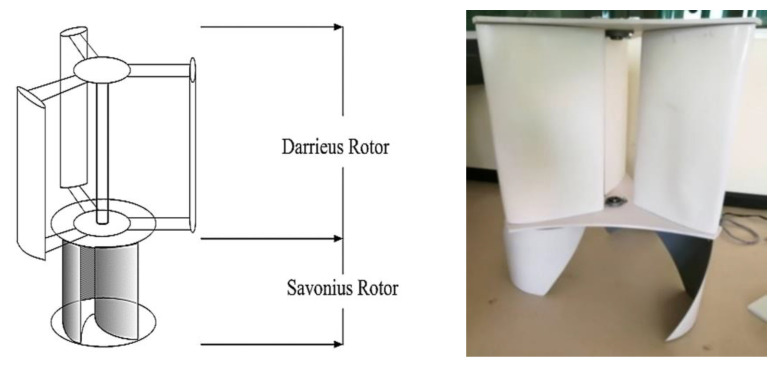
The double-rotor (Savonius and Darrieus) for wind turbine.

The experiments were conducted with S-D models, consisting of the twisted bucket at the lower section and interference of shaft at the upper section (Savonius and Darrieus; S-D Model). The wind turbine S-D rotor with two parts is obviously slightly superior to a related single-part turbine with a conventional Savonius rotor, in both power characteristics and torque. The wind turbine S-D rotor were produced from natural rubber combined with glass fiber and resin (Figure 9).

All available designs of Savonius structure, advanced NACA 0012-airfoil design, and advanced material configurations need to be applied for passive load alleviation. Accordingly, a parallel development of both strategies is advisable in the design stage, with a number of iterations before a final overall system design is arrived at. Such a process will meet the performance requirements for control, and it will be cost-effective in the long run. The blades prepared were for a vertical wind turbine with S-D rotors, operating at low wind speeds of around 2 m/s. The wind turbine blade is a composite fabricated with high quality. In this process, glass fiber is placed in an open mold and a nanocomposite is placed on the top of the mold. The liquid nanocomposite infuses into the reinforcing glass fibers thanks to the vacuum drawn through the mold. Curing and de-molding steps follow the impregnation process to complete the product, along with steps to finish the product. The components of the infusion process utilized in the work are shown in Figure 9. Three blades are formed for a wind turbine (one set) in this study. For a wind tree, six sets were manufactured. However, the double-step rotor is obviously mentioned to be slightly superior to a related single-step turbine in both power characteristics and torque.

**Figure 10:**
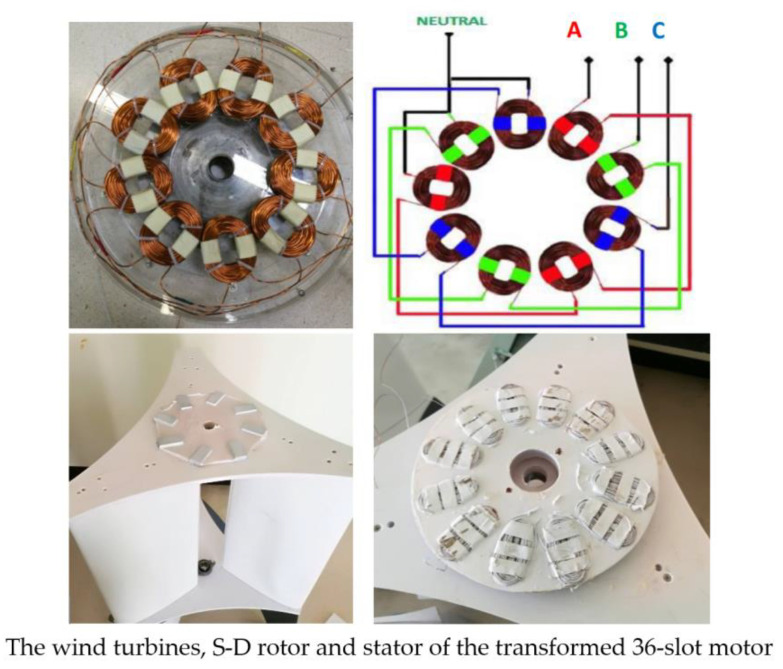
Electrical generator in wind turbine with S-D rotor: Three-phase electric power (A, B and C phase).

Figure 10 shows the structure of the rotor and the stator. The wind turbine S-D rotor and stator of the transformed 36-slot motor. An air gap of 2.5 mm was left between rotor and stator for constant reluctance to flux flowing from the rotor to the stator. Further, the magnets embedded in the rotor slots were uniformly spaced over a rotor magnet pole; the magnet had a curved shape comprised in the circular surface of the rotor which could smoothly move magnetic flux to the stator. The smoothness of the air gap helps the electromotive force waveform with low harmonic distortion. Water insulation was improved at the turbine to weather proof the device. The ball bearings were replaced with water pump bearings, and rubber strips were placed to seal possible water entrances while the coating was reinforced with paint. The original rotor motor contained the copper coil and laminate, the shaft was inserted in the center of the rotor, and the end of shaft could be connected to an assembly of blades. The commercial type neodymium magnets with arc shape were fixed on the surface of the rotor. There were 48 small surface-fixed magnets in the rotor for 8 poles attached using Loctite glue 331, giving a magnetic flux of 0.46 T/magnet path equivalent to the one created by the coil when working as a motor.

#### 3.5.2. Performance Test of Generator in the Wind Tunnel

The mixtures (100 phr epoxy resin/3 phr ENR 50/3 phr nanosilica) were successfully cured in casting molds so they had a good appearance (Table 3). The development of fibers, which are stronger than the usual glass fibers, has been carried out. The drivers of the composite material improvement include glass fibers with nanocomposite. The experiments included making wind turbine blades and examining their efficiency in a wind tunnel and in actual use. The three layers of glass fibers gave good mechanical and physical properties, and sample surfaces were smooth, shiny, and without apparent air bubble defects.

The wind tunnel was used for evaluation in a controlled simulation environment in a variety of ways. These are discrete wind speed; moving wind speed; moving power; discrete, double-power. There are two adjustable parameters for optimal operation: cut-in/cut-out thresholds (of wind speed) and test window (for average). The application of modern control design can help make predictions. The system responds on an individual system basis so as to alleviate a severe wind profile that may be building up.

**Figure 11:**
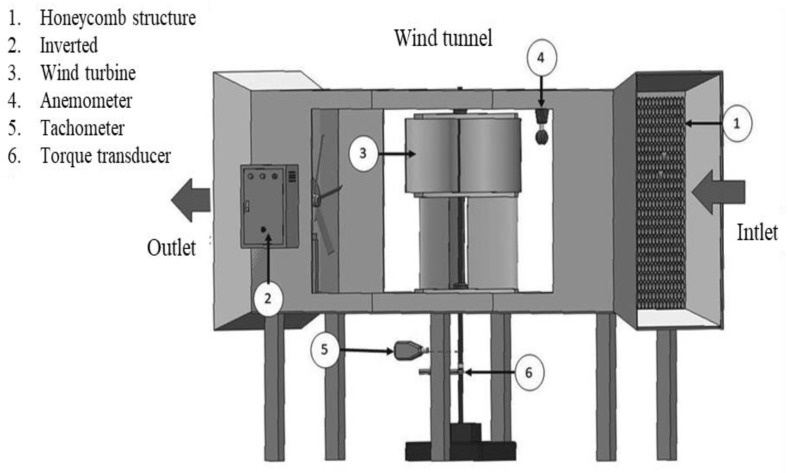
Schematic diagram of wind tunnel experimental apparatus.

The wind tunnel experiments were conducted with an open test using the wind tunnel structure made of strong sheets. The wind tunnel is driven by an inverted speed AC motor. The assessment section had a length of 2.4 m and offered a 1.2 m × 1.2 m cross-section. The inlet of the wind tunnel had a honeycomb structure installed in order to reduce turbulence and make the wind more streamlined before reaching the wind turbine. The maximum wind velocity generated could reach 30 m/s, but these experiments were carried out in the low range of wind speeds at 1.2–8.5 m/s. This investigation measured the flow field around the wind turbine by anemometer brand UNI-T model UT363 with a range of 0–30 m/s. The Digiton Photo Tachometer model DT-2234C+ with an accuracy of ±0.1 RPM was used to record the rotational speed. In the section torque of the rotor was monitored with a torque transducer, brake loads were applied to the turbine shaft through an axis connected to the torque transducer; model WSC3-030CN with the range and accuracy at 6–30 Nm, ±3% (CW). The experimental setup is shown in Figure 11.

#### 3.5.3. Data Analysis

**Figure 12:**
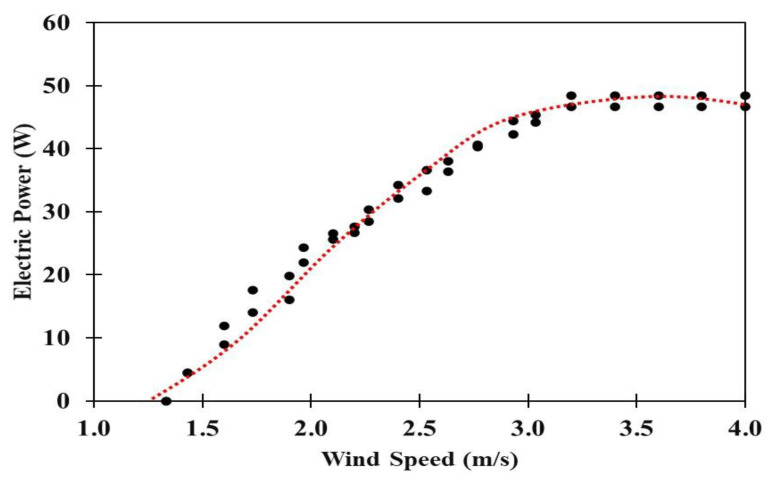
Vertical wind turbine behavior showing the relationship between electric power and rotational speed.

Figure 12 shows the real experimental wind tunnel results from measuring vertical wind turbines with blades constructed from nanocomposite blends and glass fiber. This figure shows the relationship between wind electric power and rotational speed for the S-D Model. Based on the low-speed wind turbine, the wind turbine that needs to operate at lower than 2 m/s was produced. Savonius rotor was on top of the Darrieus rotor below it, and this gave a low startup speed in the range 1.2–1.6 m/s. The best start-up at a wind speed lower than 2 m/s was produced by using lightweight materials.

Blade-fluid interaction confirms the expanded vortices downstream of the Darrieus rotor in comparison to those downstream of the Savonius rotor. This is associated with the squeezing of the vortices downstream of the Darrieus rotor due to forcing the rotor to rotate. A Darrieus turbine requires low-speed wind to produce the minimum rotational speed, while a Savonius turbine located on the top is attributed to turbine self-start at low wind speed as shown in Figure 12. The experimental results reveal that the weight of the wind turbine will become a gravitational load. The cut-in of the wind turbine increases with its weight. The positions of both the Savonius and Darrieus type rotors affected the performance of wind turbine. The Savonius was located on top of the Darrieus rotor. The rotational speed (RPM) or wind speed and torque combined from Savonius and Darrieus rotors increased with wind speed.

The Savonius-Darrieus model vertical wind turbine showed a high performance during this study. Accordingly, the Savonius was located on top of the Darrieus rotor, which allows it to start-up at low-speeds and can also increase efficiency when using lightweight nanocomposite materials.

#### 3.5.4. Economic Analysis from Power Curve for the Wind Turbine Generator

**Figure 13:**
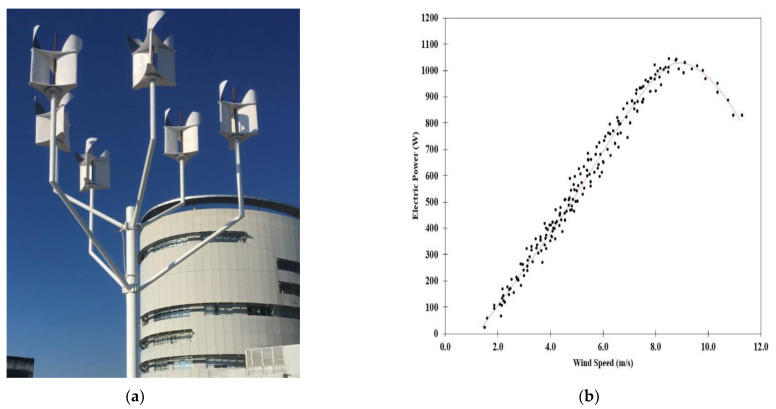
Shows the nanocomposite application in field test of a wind turbine tree system: (**a**) Savonius-Darrieus wind turbines tree, (**b**) Wind velocity (m/s).

Measured power curve for the wind turbine using the Axial Flux Permanent Magnet Generator (AFPMG), is experimentally tested for the operating speed range. The electric power was tested using lamps at the load of up to 6 kW. This wind turbine was a novel design in that by using the tree for the tower it was low cost and made of readily available materials. The properties of this AFPMG facilitate generator practical implementation of such wind power. Figure 13b shows the measured power curve for the AFPMG generator operated with wind speed at 1.2–1.6 m/s, designed for low wind speed, and with cut-off wind speed at 8.5 m/s. The wind power output of small wind turbines is used for producing electricity, and the Savonius-Darrieus wind turbine tree had six wind turbine blades per set generating up to 1000 Watts/blade. The wind power output is limited to 5 kW, which is the average from the data logger. However, electricity can still be generated at a wind speed of 2 m/s. The details of the wind turbine properties are exhibited in Table 4 and Figure 13. Wind energy is one alternative energy source in Thailand that is located near the equator and has low to moderate wind speeds that average 3–5 m/s.

According to the experiments, the S-D rotor had good performance coefficients due to its vertical axis with direct discharge flow capability. The S-D Model used twisted buckets that contributed to power coefficient increase. The twisted buckets installed on the top gave a good self-starting capability due to the twisted blade angle. A way to reduce the moment fluctuations without a significant performance loss is to use multiple stages transmitting power to the rotor shaft. Figure 13a shows a wind turbine tree that, as the velocity was increased through 4 m/s, started to rotate at 2 m/s. The pitch of the blades was varied over the range 5–30 deg to study the performance. It was found that the turbine performed best at a 20 deg pitch angle, and the startup wind speed was 2 m/s as the instantaneous cut-in wind speed. Small wind turbines are used for producing electricity, and the Savonius-Darrieus wind turbine tree had six wind turbine blades set generating up to 1 kiloWatts/blade, Figure 13b.

The results from field test of a wind turbine tree system were assessed. The blade pitch in vertical axis wind turbines was 20 deg, and the maximum wind speed tested was about 4 m/s generating approximately 5 kW. Savonius-Darrieus rotors operated well at low wind speeds below 2 m/s. Small wind turbine blades from nanocomposite materials can operate at low wind speeds. Wind turbines provide a viable alternative energy source and the vertical axis wind turbines have advantages in certain types of conditions.

The advantage of the modified-epoxy composite over the neat epoxy composite is improving the ductility of these blades for better impact resistance (higher impact strength), and, also, UV radiation and aging resistance were increased. Properties of nanocomposites for wind turbine blades should help ensure the required shape stability, and have high strength (to withstand even extreme winds, and gravity load), high fatigue resistance, low weight (to reduce the load and the effect of inertial forces), and damage resistance of the wind turbine rotor blades. In this study the blades were produced from long fiber reinforced nanocomposite laminates. In these nanocomposites, glass fibers ensure longitudinal stiffness and strength, while the epoxy resin nanocomposite is responsible for fracture toughness, out-of-plane strength and stiffness. Further work will simulate active control techniques to assess the performance capabilities of an existing structure, and indicate modifications necessary to realize further benefits.

In terms of the power generation, it is the advantage of the modified-epoxy composite over the neat epoxy composite that the volume of the power generation is higher because of impact resistance and good aging properties, and also, UV radiation resistance for long-term weathering resistance.

## 4. Conclusions

A mechanical property investigation showed that 5% ENR 50 and 3% nanosilica loading in epoxy resin improved the impact strength of the nanocomposite. The nanocomposites were evaluated for mechanical properties before and after ageing by accelerated weathering with UVB type radiation, in a weathering chamber. The tensile strength improved by 35% at 168 h of treatment. This demonstrates that the nanocomposites have good weathering resistance for outdoors use as wind turbines blades, in Savonius-Darrieus blades for low-speed wind at about 2 m/s. These nanocomposites have the high strength needed to withstand fatigue, low density to reduce inertial forces and minimize the weight, and slow degradation during use outdoors. Turbine operation could start at a low wind speed. The maximum wind speed tested was about 4 m/s generating approximately 5000 Watts. Therefore, the novel stacking of Savonius-Darrieus models in turbine while using lightweight nanocomposite materials could promote the vertical axis wind turbines for low speed operation.

## Figures and Tables

**Table 1:** Functional groups from the FTIR spectra of nanocomposite components.

Group Attributed for Peak	Wavenumber (cm^−1^)		
	Epoxy Resin	ENR 50	Nanosilica
C–O deformation	915	-	-
C–H stretching	3057	-	-
C=C–H stretching and deformation	-	837, 1588	-
C–O stretching of oxyran ring	-	873	-
H-O-H stretching mode of the absorbed water	-	-	3340, 1645
Si-O-Si asymmetric bond	-	-	1093, 788
Si-O-Si bond bending vibration	-	-	466

**Table 2:** The mechanical properties of nanocomposites.

Nanosilica (phr)	Impact Strength (kJ/m^2^)	Tensile Strength (MPa)	Hardness (Shore D)	Density (g/cm^3^)
0	11.05 ± 0.28 ^c^	27.45 ± 0.34 ^e^	78.50 ± 0.54 ^b^	0.98 ± 0.10 ^c^
1	11.42 ± 0.52 ^c^	40.43 ± 0.47 ^c^	78.60 ± 0.03 ^b^	1.02 ± 0.05 ^b^
2	11.85 ±1.30 ^c^	41.85 ± 0.14 ^b^	78.70 ± 0.83 ^b^	1.05 ± 0.15 ^b^
3	15.30 ± 0.45 ^a^	43.50 ± 0.94 ^a^	80.10 ± 0.71 ^a^	1.10 ± 0.10 ^a^
4	13.25 ± 1.15 ^b^	38.43 ± 0.92 ^d^	81.20 ± 0.53 ^a^	1.11 ± 0.05 ^a^
5	9.50 ± 1.06 ^d^	27.57 ± 0.82 ^e^	81.40 ± 0.49 ^a^	1.14 ± 0.01 ^a^

^a,b,c,d,e^ Different superscripts indicate statistically significant differences at 95% confidence by Duncan’s method. Mean ± SD: mean and standard deviation based on triplicate samples.

**Table 3:** Wind blade parts and their functions in maintaining the blade shape.

Part	Function	Materials Used
Wind blade shell	Maintaining the blade shape, resisting the wind and gravitational forces	Strong, lightweight composites (Glass fiber + epoxy resin)
Adhesive layers between composite plies, and the web and the blade shell	Ensuring the cut-off plane strength and stiffness of the blade	Strong and highly adhesive nanocomposites (Epoxy resin + ENR 50 + nanosilica)
Supported parts of the shell	Resisting the buckling load	Thickened sandwich structures with light core materials and multidirectional face laminates

**Table 4:** Technical parameters of Tree Shaped Wind Turbines.

No.	Parameter	Value
1	Wind turbine of types	Tree shaped wind turbines (TSWT)
2	Rotor of blade (radius × length)	0.4 × 1 m
3	Blade of type	Hybrid Savonius-Darrieus
4	Blade material	Glass fiber reinforced nanocomposite
5	Number of blades	3 × 6
6	Cut-in wind speed	1.2 m/s
7	Cut-off wind speed	8.5 m/s
8	Rate wind speed	6–8 m/s
9	Rate power	1 kW
10	Height of tower (m)	8 m (the tree shaped)
11	Electrical generator	Axial flux permanent magnet generator (AFPMG)
12	Number of copper coil	20#SWG
13	Phase number	3 phases
14	Pole number	8 poles

## Data Availability

The data used to support the findings of this study are available from the corresponding author upon request.
